# Spatiotemporal changes of optical signals in the somatosensory cortex of neuropathic rats after electroacupuncture stimulation

**DOI:** 10.1186/s12906-016-1510-5

**Published:** 2017-01-10

**Authors:** Myeounghoon Cha, Younbyoung Chae, Sun Joon Bai, Bae Hwan Lee

**Affiliations:** 1 Department of Physiology, Yonsei University College of Medicine, C.P.O. Box 8044, Seoul, 03722 Republic of Korea; 2 Acupuncture and Meridian Science Research Center, College of Korean Medicine, Kyung Hee University, Seoul, 02447 Republic of Korea; 3 Department of Anesthesiology and Pain medicine, Yonsei University College of Medicine, Seoul, 03722 Republic of Korea; 4 Brain Korea PLUS Project for Medical Science, Brain Research Institute, Epilepsy Research Institute, Yonsei University College of Medicine, Seoul, 03722 Republic of Korea

**Keywords:** Electroacupuncture (EA), Neuropathic pain, Primary somatosensory cortex (S1), Optical signal, Spatiotemporal change

## Abstract

**Background:**

Peripheral nerve injury causes physiological changes in primary afferent neurons. Neuropathic pain associated with peripheral nerve injuries may reflect changes in the excitability of the nervous system, including the spinothalamic tract. Current alternative medical research indicates that acupuncture stimulation has analgesic effects in various pain symptoms. However, activation changes in the somatosensory cortex of the brain by acupuncture stimulation remain poorly understood. The present study was conducted to monitor the changes in cortical excitability, using optical imaging with voltage-sensitive dye (VSD) in neuropathic rats after electroacupuncture (EA) stimulation.

**Methods:**

Male Sprague–Dawley rats were divided into three groups: control (intact), sham injury, and neuropathic pain rats. Under pentobarbital anesthesia, rats were subjected to nerve injury with tight ligation and incision of the tibial and sural nerves in the left hind paw. For optical imaging, the rats were re-anesthetized with urethane, and followed by craniotomy. The exposed primary somatosensory cortex (S1) was stained with VSD for one hour. Optical signals were recorded from the S1 cortex, before and after EA stimulation on Zusanli (ST36) and Yinlingquan (SP9).

**Results:**

After peripheral stimulation, control and sham injury rats did not show significant signal changes in the S1 cortex. However, inflamed and amplified neural activities were observed in the S1 cortex of nerve-injured rats. Furthermore, the optical signals and region of activation in the S1 cortex were reduced substantially after EA stimulation, and recovered in a time-dependent manner. The peak fluorescence intensity was significantly reduced until 90 min after EA stimulation (Pre-EA: 0.25 ± 0.04 and Post-EA 0 min: 0.01 ± 0.01), and maximum activated area was also significantly attenuated until 60 min after EA stimulation (Pre-EA: 37.2 ± 1.79 and Post-EA 0 min: 0.01 ± 0.10).

**Conclusion:**

Our results indicate that EA stimulation has inhibitory effects on excitatory neuronal signaling in the S1 cortex, caused by noxious stimulation in neuropathic pain. These findings suggest that EA stimulation warrants further study as a potential adjuvant modulation of neuropathic pain.

## Background

Neuropathic pain is defined as pain arising as a direct consequence of a lesion or disease affecting the somatosensory system [1]. It may be associated with abnormal sensations, called dysesthesia, and pain produced by non-painful stimuli (allodynia). Neuropathic pain may have continuous and/or episodic (paroxysmal) components [2, 3]. Moreover, the state of chronic pain induced by neuropathic pain are related to psychological depression, unpleasant sensations, and discomfort which reduce the patients’ quality of life [2, 3]. Although various medications have been investigated for neuropathy treatment, the management of neuropathic pain still needs improvement [4, 5].

Today, neural disorder management by acupuncture stimulation are widely adopted [6, 7]. The advantages of acupuncture stimulation include minimized side effects of pharmaceutical or other treatment methods, lower aversion in patients, and affordable costs [8]. Numerous studies have attempted to elucidate the pathophysiological mechanisms of acupuncture effects on various chronic pain symptoms in both patients and experimental animal models [9–11]. In order to identify the effects of acupuncture treatment, previous studies observed changes in blood pressure, activity changes of endogenous opioids within the central nervous system (CNS) [12], and brain activation by, using advanced technologies such as electroencephalogram [13], functional magnetic resonance imaging (fMRI) [14], and positron emission tomography [15]. Despite these efforts, the mechanisms of acupuncture-induced analgesia remain unclear [11, 16].

In our previous study, we observed the distinctive activation pattern of optical signals in the primary somatosensory cortex, after electrical stimulation of the hind paw in neuropathic rats [17]. The ability to image the spatiotemporal cortical activation pattern is beneficial for understanding the mechanisms of neural plasticity such as formation, progression, and extension. Current optical imaging studies show a high spatial resolution for neural activities in the cerebral cortex, visualize temporal patterns of activated signals in the cortical tissue, and investigate the characteristic patterns in neural activation of the brain [18–20]. In a recent study regarding spatiotemporal patterns of activated signals using optical imaging, Lusting et al. [19] reported a spatial shift in the center of activation in the somatosensory cortex within a 10 to 20-ms period after whisker stimulation. In addition, spatiotemporal dynamics of long-term potentiation (LTP) in the insular cortex (IC) was also reported. Mizoguchi et al. [20] found that the excitatory propagations, after LTP induction, were spread preferentially towards the rostral IC area.

In order to investigate the analgesic effects of electroacupuncture (EA), spatiotemporal changes of neural excitability in the S1 cortex were compared in control (intact), sham injury, and nerve-injured rats after peripheral stimulation. This experiment verified the responsive area and intensity changes in the S1 cortex after electrical stimulation of the hind paw. Secondly, neural excitabilities were compared, before and after electroacupuncture (EA) stimulation, in order to evaluate the analgesic effects of EA stimulation on neuropathic pain. For EA, well-known analgesic acupoints, Zusanli (ST36) and Yinlingquan (SP9), were used. The spatial activity maps of optical signals, maximal spread of optical activity, and time-dependent changes in optical activity were analyzed after EA stimulation.

## Methods

### Experimental animals and neuropathic surgery

All animal experiments were performed in accordance with protocols approved by the Institutional Animal Care and Use Committee of Yonsei University Health System. All efforts were made to minimize animal suffering and to reduce the number of animals used.

Male Sprague–Dawley rats (Koatech, Pyeongtaek, Gyeonggi, Korea; 220–250 g) were anesthetized with sodium pentobarbital (50 mg/kg, i.p.). A segment of the sciatic nerve of the left hind limb, between the mid-thigh and the popliteal fossa, was exposed by a skin incision and blunt dissection through the biceps femoris muscle. The three major divisions of the sciatic nerve (tibial, sural, and common peroneal nerves) were clearly separated, based on individual perineurium. Tibial and sural nerves were tightly ligated and then transected, meanwhile common peroneal nerve was left intact. Complete hemostasis was confirmed, and the wound was closed with muscle and skin sutures [21]. Control rats remained intact, and sham injury models were operated without nerve injury.

### Behavioral tests

For confirmation of pain development following surgery, behavioral tests were performed on post-operative days 1, 4, 7, and 14. Two components of neuropathic pain (mechanical allodynia and cold allodynia) were assessed in all rats. To measure mechanical allodynia, rats were placed on a metal mesh floor under a custom-made transparent plastic dome (8 × 8 × 18 cm). Innocuous mechanical stimuli were applied to the sensitive area of the nerve-injured hind paw with a von Frey filament every three to four seconds (8 mN bending force, ten repetitions). The frequency of the hind paw withdrawal, out of 10 trials, was expressed as a percentage (response rate (%) = number of hind paw withdrawals / number of trials × 100). To quantify cold sensitivity, we observed the withdrawal response to acetone that was applied to each hind paw every five minutes (five repetitions). The frequency of hind paw withdrawals, out of five trials of acetone application, was also expressed as a percentage. After the behavioral test (at 14 days after surgery), animals were subjected for optical imaging.

### Surgery for optical recording

**Fig. 1:**
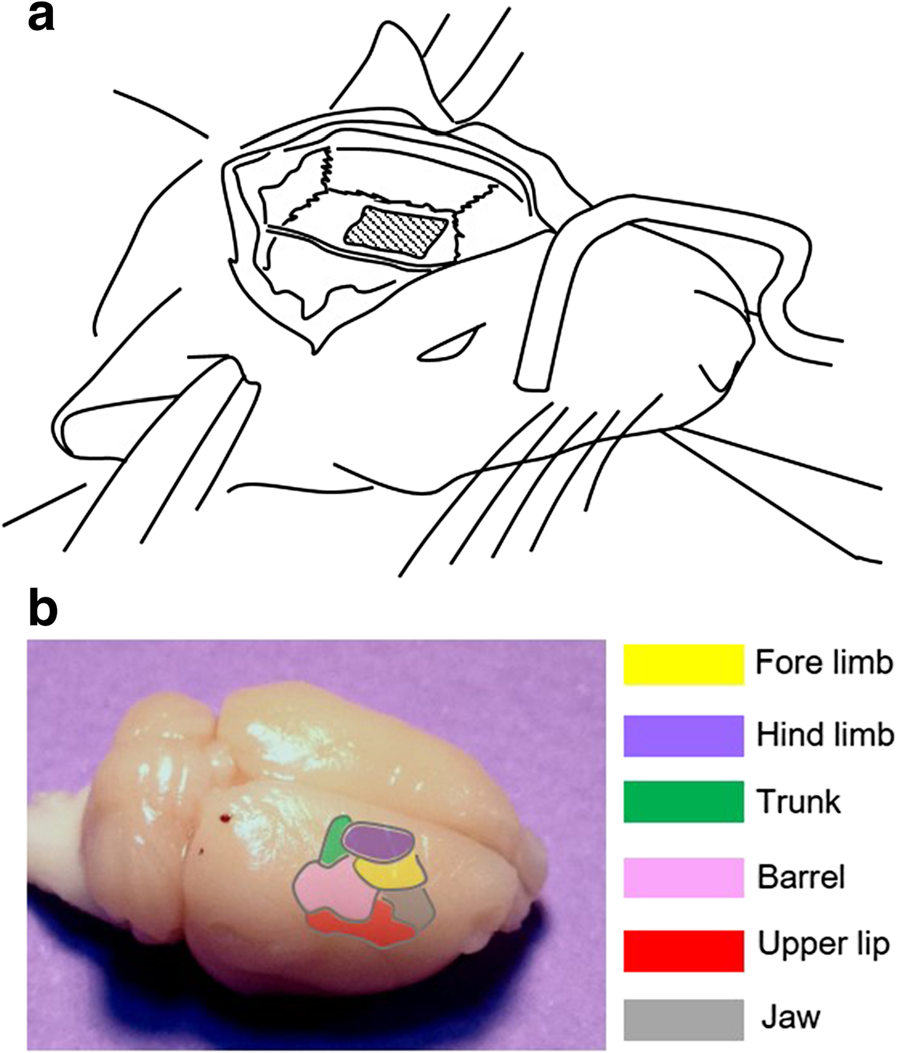
Imaging regions of primary somatosensory cortex (S1) and surrounding cortices. **a** A schematic drawing of the preparation for optical imaging of the S1. **b** A template outlining each subdivision in dorsal cortices

Two weeks after nerve injury, rats were re-anesthetized with urethane (1.25 g/kg, i.p.). Trachea was cannulated for artificial respiration during optical imaging. Electrocardiography was monitored, and body temperature was maintained at 37 °C with a heating pad (Harvard Apparatus, South Natick, MA, USA). Craniotomy was performed under the deep sedation. Vecuronium bromide (0.2 mg/kg; Huons Co., Hwaseong, Korea) was injected for muscle relaxation, and then rats were artificially ventilated using an animal respirator (Harvard Apparatus, South Natick, MA, USA) to exclude the influence of respiratory movements during optical recording. The animals were placed in a stereotaxic frame (Narishige Scientific Instrument Laboratory, Setagaya, Tokyo, Japan) to stabilize the head during the optical imaging experiment. A cranial window (10 X 5 mm) was made over the somatosensory cortex, contralateral to the nerve injury. A well of dental acrylic was built around the exposed cortex. Then, dura of the exposed cortex was removed (Fig. 1). The surface of the cortex was exposed to the voltage-sensitive dye (VSD; di-2-ANEPEQ, 50 μg/mL in saline, Molecular Probes, Eugene, OR, USA) for one hour. Then, the cortex was rinsed twice with saline and kept moist.

### Optical recording

Recording surface was placed under the optical microscope (Leica Microsystems Ltd., Heerbrugg, Switzerland), equipped with a 1x lens and halogen lamp (150 W). Light from the filtered tungsten-halogen lamp (480–550 nm) was reflected onto the cortical surface via a dichroic mirror. Fluorescence images were acquired through an absorption filter with a rate of 3.7 ms/frame (MiCAM02, BrainVision, Tokyo, Japan). The CCD-based camera captured a 4 x 3 mm^2^ imaging area, consisting of 184 x 124 pixels. For the electrical stimulation, a bipolar electrode with a 5 mm tip interval was used. Twenty consecutive images in response to electrical stimulation of the nerve-injured hind paw (20 trials = about 40 s recording, 200 ms delay, 0.1 ms pulse width, 2 s interstimulus intervals, 0.6 mA intensity) were averaged to reduce artifacts, including interference of heartbeats, mechanical vibration after breathing, and spontaneous neural activity. Image acquisition was triggered by an electrocardiogram, using a stimulus/non-stimulus subtraction method [22, 23].

### Electroacupuncture (EA) stimulation

For EA experiment, two stainless-steel acupuncture needles (0.25 mm in diameter and 4 cm in length) were inserted into the ST36 and SP9 acupoints in neuropathic rats. One acupuncture needle was inserted into the ST36 and the other was inserted into the SP9. The ST36 point is located near the knee joint, 5 mm lateral to the anterior tubercle of the tibia. The SP9 point is located between the posterior border of the tibia and the gastrocnemius muscle, which is near the knee joint on the inferior border of the medial condyle of the tibia. Stimulation at these points is known to produce an analgesic effect for various types of pain symptoms, including neuropathic pain. For EA stimulation, train-pulses (2 Hz, 0.1 ms pulse width, 0.6 mA) were applied to the needles inserted into acupoints for 10 min, using an electrical stimulator (A385, WPI, Sarasota, FL, USA). Afterwards, changes in the fluorescence activity of S1 cortex were observed at each time point (0, 30, 60, 90, 120, and 150 min) after EA. In order to quantify EA stimulation effects on neuropathic pain, EA stimulation were applied at sham EA acupoints (locations that are not known as acupuncture points) on neuropathic pain rats. In addition, the needle insertion effects without electric stimulation were quantified using the same acupoints at pre- and post-needle insertion.

### Analysis of optical signals

Acquired optical images were processed using BV analysis software (Brain Vision, Tokyo, Japan). First, the values of all pixels in images are divided by F_max_ for standardization. Pixel intensities of less than 30%, over the maximum change in optical activities, were removed to minimize background signals. The images were processed with a spatial filter (for replacement with the averaged value) and cubic filter (for 2D and temporal filter processing). Then, the fluorescence activity patterns were captured at time points of peak activation, for each stimulation point. For standardization, activated areas were analyzed in terms of changes in fluorescence (ΔF/F; i.e., the value of all pixels divided by the brightest value). The ΔF/F values in activated regions were calculated and then compared. To analyze the activated pattern and area, activation map function of BV analysis software was used. To analyze the activated area, each color image was converted to a monocolor image. The converted area in each captured image was calculated and expressed as a percentage of the area of activation compared to the entire captured area (activated area/whole captured area x 100). In addition, the MetaMorph program (Universal Imaging Co., Downingtown, PA, USA) was used to convert raw data and to analyze the measured activation area.

### Statistical analysis

Data were presented as the mean ± standard error of the mean (SEM). Behavioral changes of withdrawal rate were analyzed using two-way analysis of variance (ANOVA), followed by Dunnett’s post-hoc pairwise comparisons. Differences in intensities of optical signals and activated areas were analyzed using one-way ANOVA, followed by Dunnett’s post-hoc pairwise comparisons. A *p*-value less than 0.05 was considered significant.

## Results

### Development of mechanical and cold allodynia

**Fig. 2:**
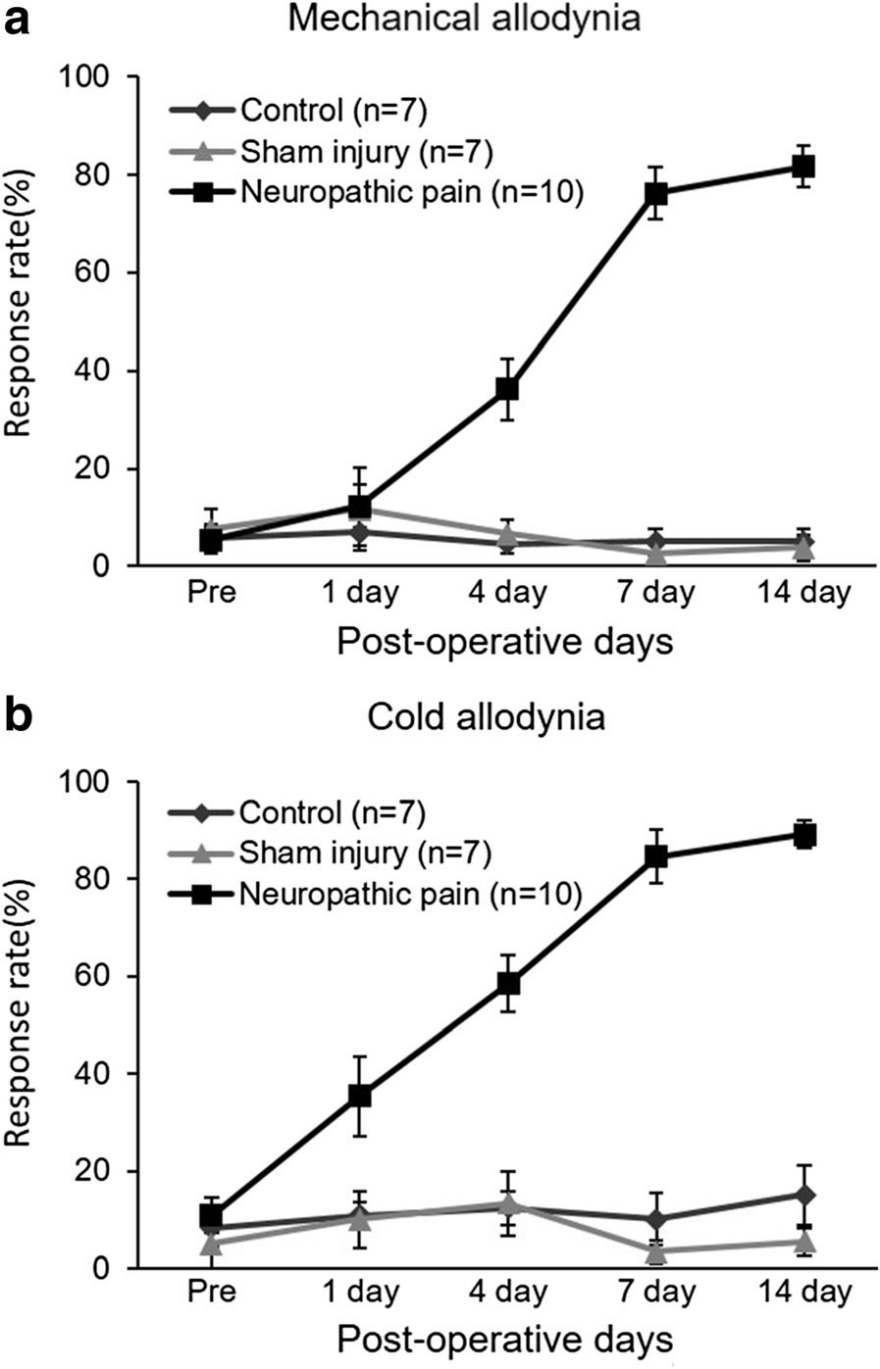
Development of mechanical (**a**) and cold allodynia (**b**) after nerve injury. Control(intact): group without surgery; Sham injury: sham-operated group; Neuropathic pain: nerve injury group. Values are represented as mean ± SEM, and response rate is indicated as a percentage (number of foot withdrawals/number of trials × 100)

Behavioral signs of neuropathic pain were produced after nerve injury (Fig. 2). Fig. 2a and b shows the development of mechanical allodynia and cold allodynia, respectively. Two-way ANOVA showed significant effects in between groups and at different time points. Dunnett’s post-hoc multiple comparisons showed that the neuropathic group displayed significantly higher withdrawal responses to von Frey filament from four days after nerve injury (P < 0.05) and to cold stimulation from one day after nerve injury (P < 0.05). These behavioral results indicated that nerve-injured rats were more sensitive to mechanical and thermal peripheral stimulations, and hypersensitivity has gradually developed.

### Spatiotemporal pattern of optical signals in response to noxious stimulation

**Fig. 3:**
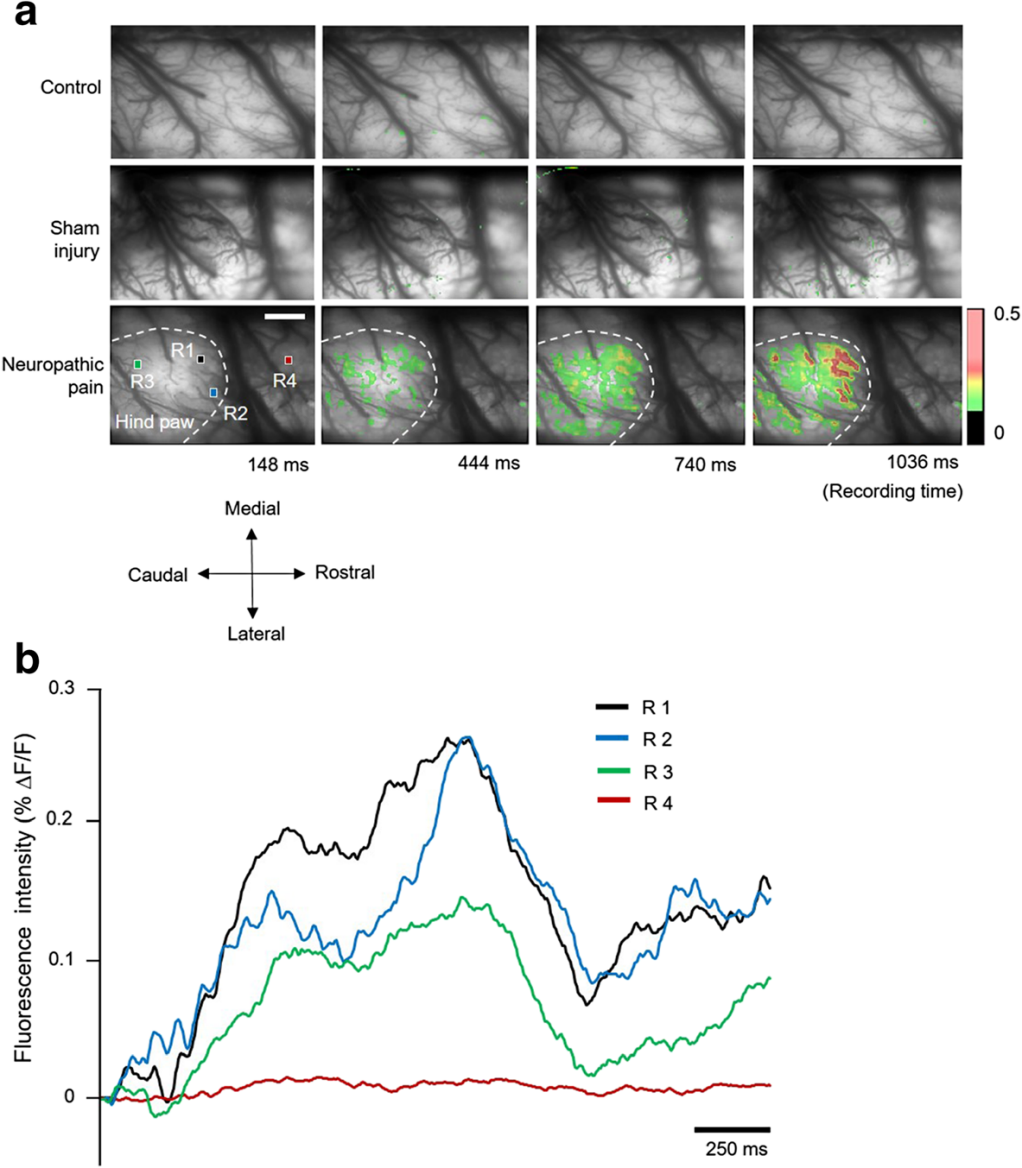
Spatiotemporal neural activity after electrical stimulation of the receptive field. **a** Four time-dependent images in each row show the change in neural activities and propagated activated area at each time point. Upper row indicates the S1 cortex in the normal control rat brains. Middle and bottom rows show the S1 regions of sham surgery and nerve-injured rats, respectively. Time-dependent fluorescence activities and activated areas were observed and analyzed (scale bar = 800 μm). **b** Each optical signal was observed from four colored squares in A (R1: *Black*, R2: *Blue*, R3: *Green*, and R4: *Red*). After electrical stimulation, neural activities reflected by fluorescence showed immediate changes. However, there was less activity signal changes recorded at the red square (outside of the activated area) (scale bar = 250 ms)

Peripheral electrical stimulation was applied to the left hind paw (ipsilateral side of neuropathic surgery) through a stimulation electrode, in order to confirm the cortical excitation in S1. Following the methods developed in our previous study [17], stimulus intensity was set at 0.6 mA to record constant fluorescence changes. In order to compare the cortical activation levels in S1 areas of control (intact), sham injury, and neuropathic rats, electrical stimulation was applied to the hind paw contralateral to the optical window. Following 0.6-mA electrical stimulation, differences in activated signals in the S1 cortex of control and sham operation rats were not significant (Fig. 3a upper and middle rows). However, in nerve-injured rats, color-changed and propagated optical signals were observed in the S1 cortex, after electrical stimulation of the hind paw (Fig. 3a bottom row). Stimulus-evoked neural activities were expressed only in a restricted area of S1. Initial optical signals started at 220 to 250 ms, after peripheral stimulation in nerve-injured rats (Fig. 3b). After the enlarged and propagated optical signals were maintained for up to 1200 ms, incomplete and weaker signals were observed. To quantify spatiotemporal profiles of fluorescence activities, fractional changes (ΔF/F) were plotted as wave forms (Fig. 3b). In Fig. 3b, each colored line indicates spatiotemporal activities of the fluorescence responses, produced by electrical stimulation of the hind paw over the entire recording time. Each colored line (black, blue, green, or red) is collated with the corresponding colored square (black, blue, green, or red) in Fig. 3a (bottom row), and shows the neural activities reflected in fluorescence. However, the red-colored square (R4), out of interest region, shows weaker signal activity during the recording period.

### Changes in optical signals after EA stimulation

**Fig. 4:**
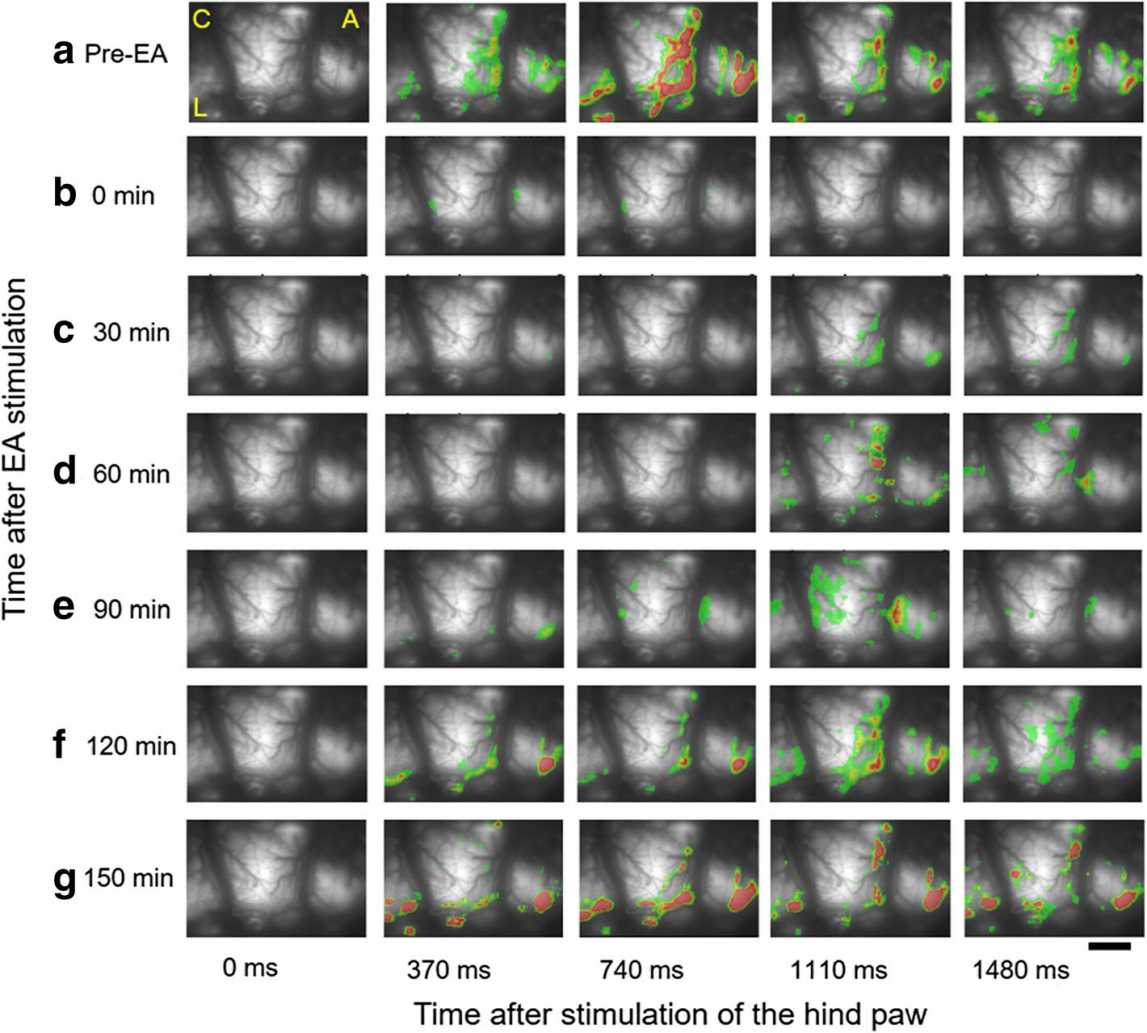
Spatiotemporal activity maps of optical activity during recording time. The five images in each row indicate frames consisting of every 100^th^ frame in recording time. **a** Following electrical stimulation of the receptive field before EA in nerve-injured rats, significantly activated signals were detected. **b** Following electrical stimulation of the receptive field immediately after EA, intensity and activated area of fluorescence were typically reduced. **c**, **d**, and **e** Reduced and weak signals were observed 30, 60, and 90 min after EA. **f** and **g** By 120 and 150 min after EA, optical signals gradually recovered (C: caudal, A: anterior and L: lateral, scale bar = 800 μm)

As a measure of changes in neural activity following EA stimulation, optical images were recorded before EA stimulation and then at 0, 30, 60, 90, 120, and 150 min after EA stimulation. Five images in each row of Fig. 4 indicate fluorescence changes at each recording time. Every 100^th^ frame was captured and collected, for the comparison of neural responses to EA stimulation at each time point. In the first row of pre-EA stimulation, we observed responses of increased and propagated neural activities after peripheral electrical stimulation (Fig. 4a). However, immediately after EA stimulation, the activated signals and areas were typically reduced (Fig. 4b). In addition, the reduced neuronal activities and decreased activated cortical areas did not recover until 90 min after EA stimulation (Fig. 4c, d and e). Time-dependent recovery of neuronal activity in the S1 area was observed, at 120 and 150 min after EA stimulation (Fig. 4f and g).

### Changes in fluorescence intensity and activated area after EA stimulation

**Fig. 5:**
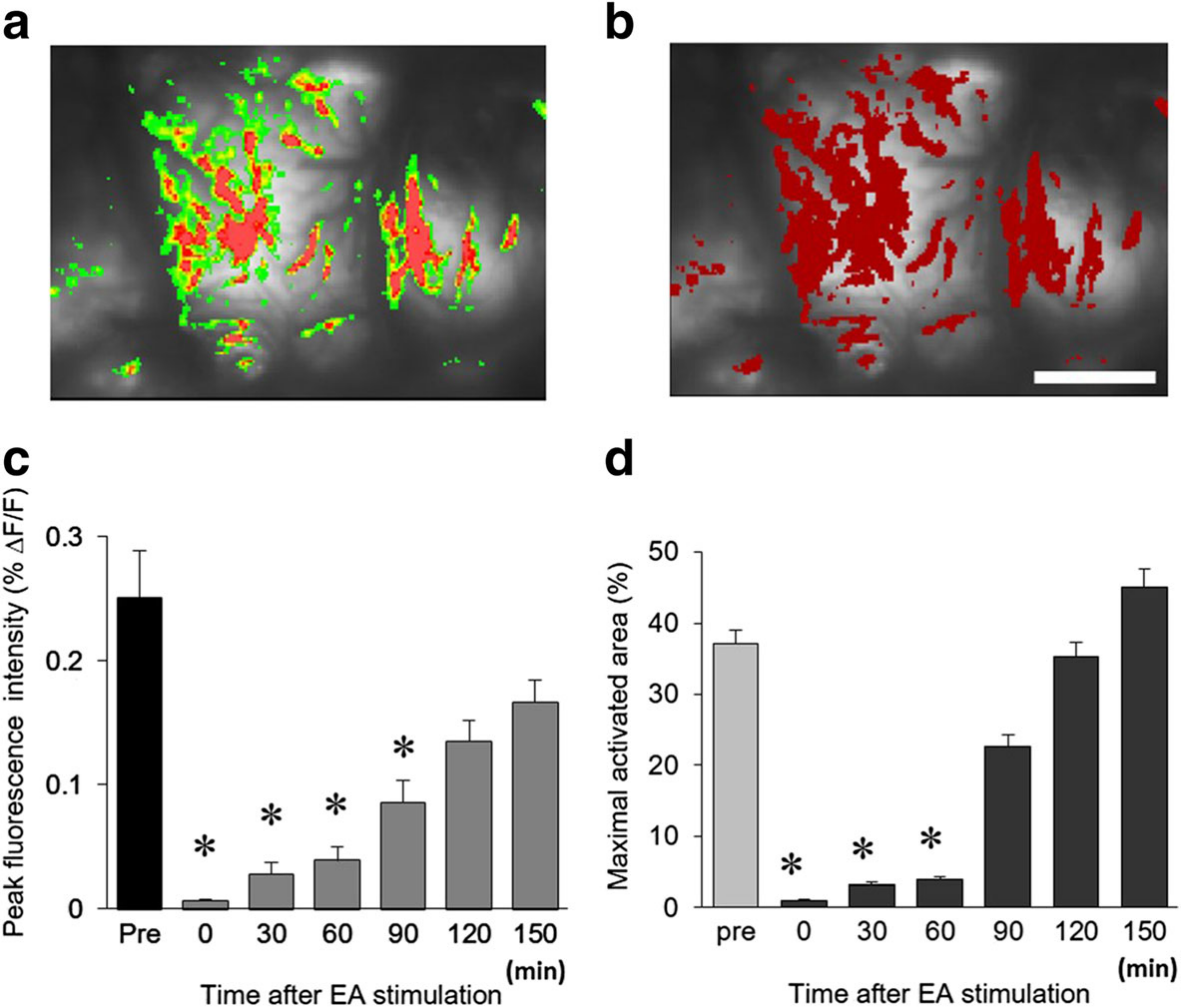
Changes of fluorescence intensity and activated area after EA stimulation. **a** Activated optical signals had different colors (*red*, *yellow*, and *green*). **b** Using the MetaMorph program, images were converted from colorful images to singular (*red*) color images (scale bar, 800 μm). Then activated area were calculated in the captured S1 cortex. **c** Each activated signal in pre- or post-EA stimulation was detected after electrical stimulation of the hind paw in nerve-injured rats. The average peak fluorescence is shown. After EA stimulation, the activated fluorescence signals became typically smaller and reduced. **d** Time-dependent changes in the maximal activated area in S1 cortex. Changes of fluorescence area at pre- and post-EA stimulations are presented (*P* < 0.05, *n* = 10)

The alteration of cortical activation patterns was measured from 0 to 150 min after EA stimulation. Typical optical images and their corresponding activated areas are presented in Fig. 5a and b. Changes of fluorescence intensity at different time points were analyzed and drawn in Fig. 5c. The results indicate that fluorescence intensities were reduced immediately after EA stimulation, and that the reduction lasted until 90 min after EA stimulation. The peak fluorescence intensities were recovered time-dependently (*P* < 0.05).

Figure 5b shows transferred images from optical signals to a single color activated map in the S1 cortex. The comparison of maximal activated areas, of pre- and post-EA stimulation in the S1 area, were calculated and graphed in Fig. 5d. Activated areas of the S1 cortex were significantly reduced at 0, 30, and 60 min, but not at 90, 120, and 150 min after EA stimulation (*P* < 0.05).

**Fig. 6:**
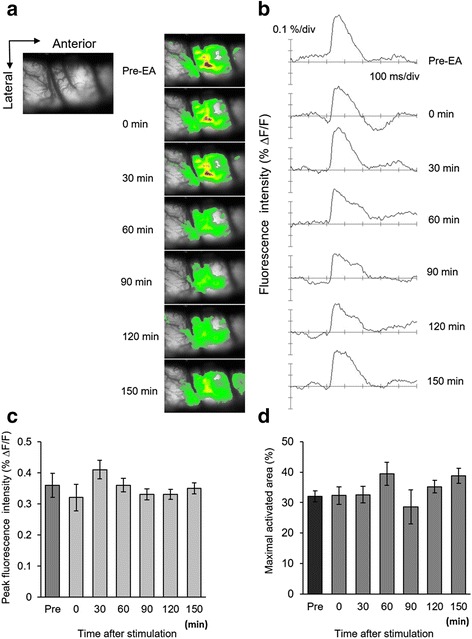
Spatiotemporal activities of sham EA stimulation in neuropathic rats. Sham EA stimulation was characterized by EA stimulation at nonacupoint locations (**a**) Each activated signal in pre- or post-sham EA stimulation was detected after electrical stimulation of the hind paw in nerve-injured rats. After sham EA stimulation, the enlarged and propagated activated area did not reduce. **b** The peak activated fluorescence signals did not change after sham EA stimulation. **c** The average of peak fluorescence has shown continuance activated pattern. **d** Time-dependent observed maximal activated area in S1 cortex is presented (*n* = 5)

In order to investigate the neuronal excitability and inhibitory effect of neuronal excitation by actual EA stimulation, spatiotemporal changes of neural excitability in the S1 cortex, after peripheral stimulation, were compared with changes following sham EA stimulation. Sham (needle insertion at nonacupoint locations) EA stimulation was applied in nerve-injured rats. Figure 6 shows the spatiotemporal fluorescence activities of the S1 area, following hind paw electric stimulation after sham EA stimulation. After electrical stimulation of the hind paw, activities were observed by the fluorescence in the stimulus-related S1 area, from 0 to 700 ms (Fig. 6b). In comparison of activities following real EA stimulation, spatiotemporal activities did not diminish after sham EA stimulation. Analogous optical activities were observed until 150 min after peripheral stimulation.

Changes in peak fluorescence intensity were measured at different time points and drawn in Fig. 6c. Fluorescence intensities were compared by pre- and post-sham EA stimulation. However, optical signals in the S1 cortex were not significantly reduced after sham EA stimulation. These results indicate that the sham EA stimulation had no effect on nerve injury-induced hyperalgesia. Additionally, the comparison of maximal activated areas, before and after sham EA stimulation in the S1 area, were graphed in Fig. 6d. Activated areas were not attenuated after sham EA stimulation (*P* > 0.05). Activated areas of S1 tended to be reduced at 90 min, although they were not statistically significant (*P* > 0.05).

**Fig. 7:**
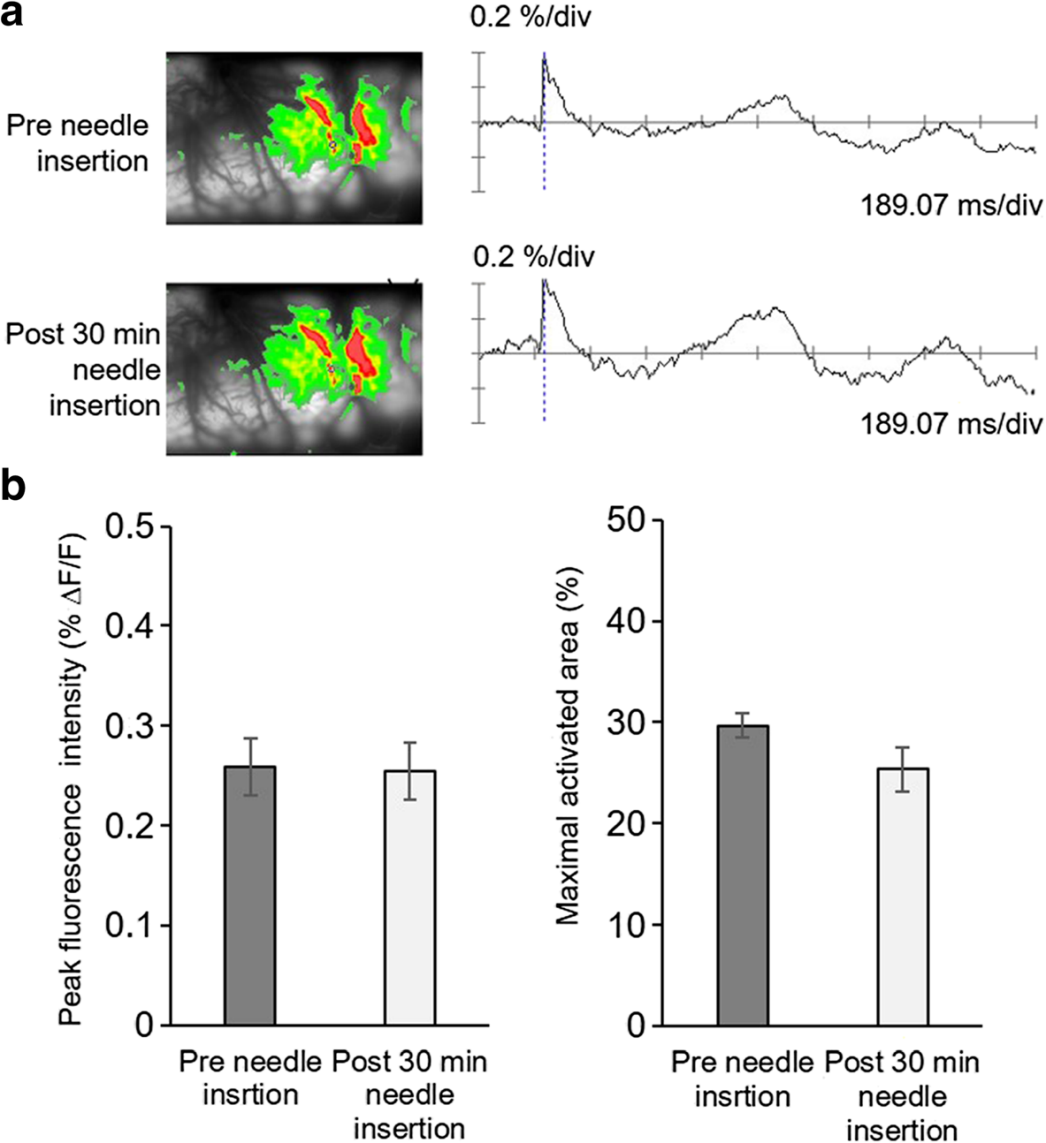
Effects of the pre- and post-needle insertions on spatiotemporal fluorescence activities. **a** The peak activated fluorescence signals were not changed between pre- and post-needle insertions. **b** The maximal activated areas in S1 cortex were compared. Maximal activated areas between pre- and post-needle insertions were not significantly different (*n* = 4)

In addition, changes of neural excitability in the S1 cortex were compared at two different time points (Pre-needle insertion and post 30 min needle insertion) for the needle insertion effects without electrical stimulation. Figure 7 shows the comparison of spatiotemporal fluorescence activities of pre- and 30 min after needle insertion time point. Although needles were inserted at ST36 and SP9 acupoints without electrical stimulation, comparable fluorescence activities were observed in the stimulus-related S1 area at pre- and post-needle insertion time point (Fig. 7a). Peak fluorescence intensities were compared with pre- and post-needle insertion (Fig. 7b, left). However, peak fluorescence intensities in the S1 cortex between pre- and post-needle insertion did not changed after needle insertion. In addition, comparison of maximal activated areas at pre- and post-needle insertion time points were not decrease after needle insertion (Fig. 7b, right) (*P* > 0.05).

### Propagation of optical signals

**Fig. 8:**
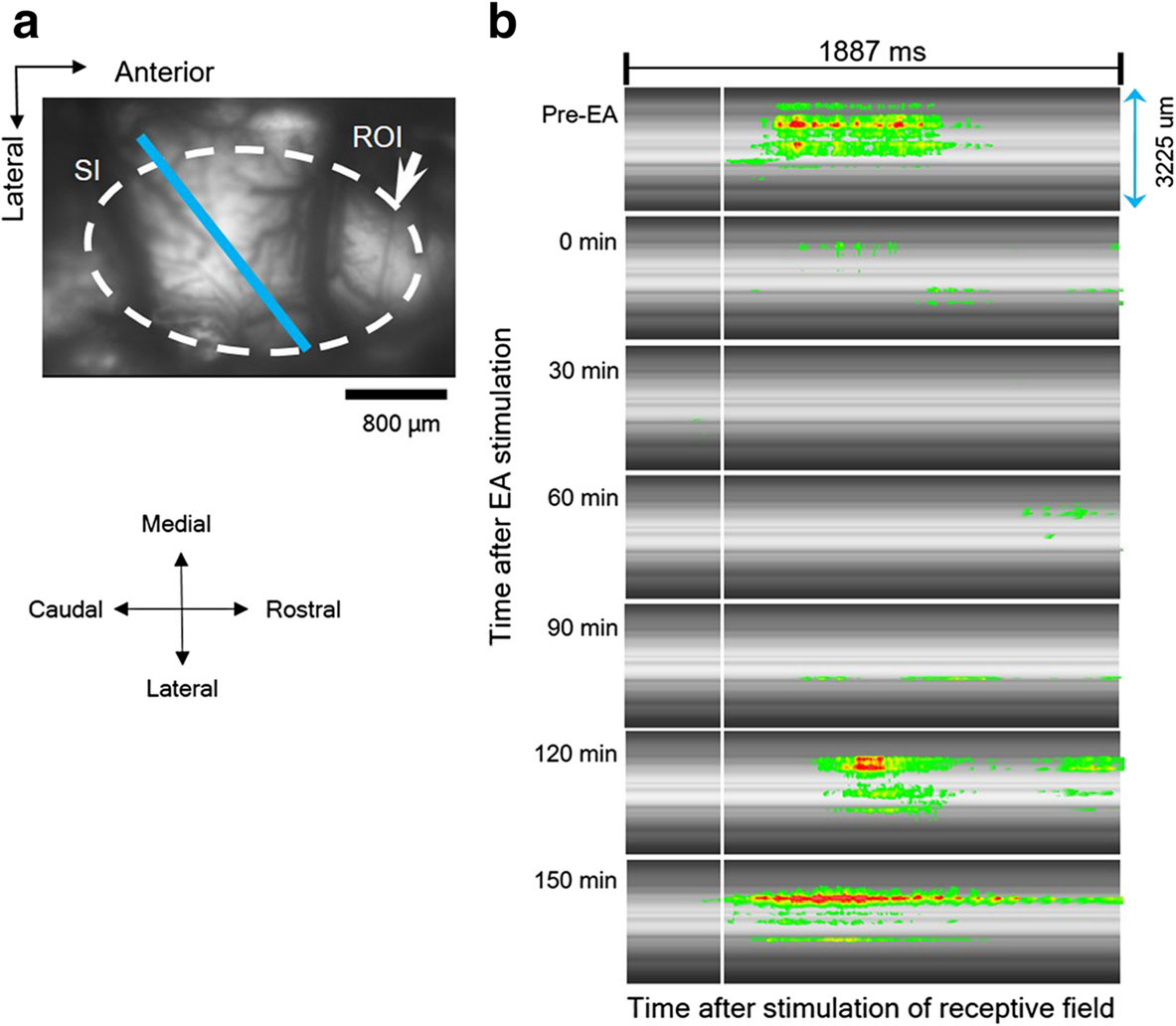
Spatiotemporal activities in S1 cortex. **a** The dashed *white circle* outlines the maximal spatial boundaries of optical activity in the S1 cortex area. A *bold blue* line is used for spatiotemporal analysis in the S1 region, the region of interest (ROI) borderline of neural excitation area; scale bar = 800 μm). **b** Spatiotemporal map of the optical activity during data acquisition. Each vertical white line represents the time point of electrical stimulation of the hind paw of nerve-injured rat. Before EA stimulation (Pre-EA), regions of neural activity were presented by distinctive fluorescence signals during recording time. In contrast, after EA stimulation, neural activity immediately decreased and then time-dependently recovered until 150 min

In nerve-injured rats, proliferation of fluorescence signals was observed in the pain-related S1 area after electrical stimulation of the hind paw. Figure 8 shows the propagation of activated optical signals. In Fig. 8a, the dashed white circle outlines the spatial boundaries of optical activation in the cerebral cortex by peripheral stimulation. For the comparison of spatiotemporal patterns at pre- and post-EA stimulation, optical signals were obtained along the designated solid blue line in the region of interest (ROI) in Fig. 8a (from 0 to 1887 ms in Fig. 8b). In Fig. 8b, each row indicates a different recording time, and the vertical white line represents the time point of electrical stimulation of the hind paw. The bilateral arrow (3225 μm length) in Fig. 8b indicates the length of blue line in Fig. 8a. The first row in Fig. 8b shows wider and longer activation signals with the solid blue line in the ROI. By contrast, the second row, which was recorded at 0 min after EA stimulation, shows drastically decreased fluorescence signals. Time-dependent recovery of neural activities was recorded until 150 min after EA stimulation.

## Discussion

This study examined the neural excitation of neuropathic rats and the inhibitory effects of EA stimulation. Neuronal activation patterns in neuropathic rats were observed in order to investigate neuroplasticity after nerve injury. The increased amplitudes of optical signals in the S1 cortex were recorded, following electrical stimulation of the peripheral receptive field. In this regard, the optically mapped cortical activity, due to electrical stimulation in neuropathic rats, was amplified and expanded. In addition, the decline of neural activities induced by EA was also observed. These findings imply that EA has inhibitory effects on painful neuropathic symptoms.

### Spatial profile of excitatory propagation in the neuropathic rat

In the present study, amplified and expanded neuronal signals, followed by hind paw stimulation in nerve-injured rats, may provide overt evidence that persistent hyperalgesia and allodynia in neuropathic rats give rise to alteration in the pain matrix of the brain [17, 18]. fMRI studies of hyperalgesia in rats have demonstrated the involvement of the somatosensory cortex in the pain pathway originating from nociceptive inputs [24, 25]. Such occurrence leads to additional sensitization processes in the brain, known as cerebral sensitization [26]. A recent study on membrane potentials found that the dye-related optical signals represented the integral changes of membrane potential in neurons, as well as possible contributions from the depolarization of neighboring glial cells, such as oligodendrocytes, astrocytes, and microglia [27]. Here, we compared the neuronal activity changes in S1 cortices of control, sham injury, and neuropathic rats. Previous results of stimulus-dependent increased optical signals, [17] as well as results from the present study, strongly suggest that elevated and extended neural signals in the S1 cortex after electrical stimulation could explain the increased depolarization of neurons in the cortex of nerve-injured rats. Although the S1 area directly received painful sensation through the ascending nociceptive pathway, the enlarged activation pattern of the S1 region could not be fully explained by pain perception. However, the present results may offer an idea of the neuronal excitation of neuropathy, as well as the mechanisms of neural plasticity.

### Reduction in excitatory neural activities after EA

In our previous study, we reported that neuronal responses to EA stimulation can be visualized in naïve rat primary somatosensory cortex using an optical imaging system [28]. As shown in the present study, propagated and enlarged neural activities in the S1 area of neuropathic rats were inhibited immediately after EA stimulation. EA-induced analgesic effects lasted up to 120 min after EA. Our previous study [29] reported that the pain-relieving effect of EA on mechanical allodynia lasted for three hours. Taken together, these data proposed that EA stimulation has pain-relieving effects on neuropathic pain.

EA stimulation reports showed the analgesic effects of EA in the brain [14, 30, 31]. After EA stimulation, higher levels of brain metabolism were observed in the middle temporal cortex, orbital frontal cortex, insula, middle frontal gyrus, angular gyrus, post-cingulate cortex, precuneus, and middle cingulate cortex [30].

EA stimulation primarily induces activity in the somatosensory related region, including those in the inferior parietal lobule; secondary somatosensory cortex; insular cortex; and thalamus, except for the superior temporal gyrus [31]. Moreover, EA inhibition of hyperalgesia is involved in the release of mu and delta receptor agonists [32]. Therefore, it is plausible that EA may activate norepinephrinergic neurons in the locus coeruleus that descend to the spinal cord, to activate the opioid system while inhibiting Fos expression and hyperalgesia [33]. These data suggest that EA activated the supraspinal norepinephrine-containing neurons and increased the spinal release of opioids, to alleviate hyperalgesia in the rat model of pain. Collectively, EA stimulation may act on pain modulation by activating the noradrenergic descending inhibitory pathways. A similar activation was seen in neurotropin-caused pain inhibition, in an adjuvant-induced rat model of arthritis [34].

Although we did not analyze serotonin and norepinephrine after EA, our findings strongly support the idea that the pain inhibition phenomena in the spinal cord contribute to the reduction of neuronal excitability in the sensory cortex, after EA stimulation in neuropathic pain.

In the present study, we applied EA stimulation to the ST36 and SP9, and observed an immediate reduction of neuronal activity in the cerebral cortex. Our study provides direct evidence that EA inhibits neuronal excitation in the S1 cortex, after noxious peripheral stimulation. Furthermore, our results indicated that EA stimulation can modulate neuropathic pain.

### Mechanism of EA-produced analgesia in neuropathic pain

In our previous reports, mechanical and cold allodynia responses significantly decreased after acupuncture or EA stimulation [29, 35, 36]. Zusanli and Yinlingquan are most frequently used of acupuncture points and they are certainly the most intensively studied treatment in acumoxa therapy. In our previous report [35], we performed an experiment related to analgesic effects of manual acupuncture at Zusanli (ST36) and Yinlingquan (SP9) acupunctoints. As results, acupuncture stimulation significantly reduced mechanical and cold allodynia up to 210 min after acupuncture stimulation [35]. In addition, we observed changes in withdrawal responses after EA stimulation (ST36 and SP9) in neuropathic rat. As a result, EA stimulation group showed more meaningfully reduced withdrawal response. [29, 36]. EA inhibited the expression levels of nitric oxide (NO) synthase in the spinal cord of neuropathic rats [29]. Dorsal root activities also declined after acupuncture stimulation at ST36 and SP9 [35]. In addition, inhibited NO synthesis and reduced expression of proinflammatory cytokines, such as IL-6, IL-1β, and TNF-α, were observed after EA stimulation [4, 7]. Multiple lines of evidence on EA-induced analgesia suggest that EA stimulation mediates the release of neurotransmitters at several sites in the CNS. EA studies have investigated the mechanisms of analgesic effects of EA stimulation and showed involvement of opioids [37], norepinephrine [38], serotonin [39], glutamate and its receptors [40, 41], glial cells [42], and cytokines [36] in both peripheral and spinal sites. Overall, EA-induced bioactive chemicals are involved in the inhibition of neuropathic pain [43].

Earlier studies suggest that the hypothalamus, having the most abundant endorphinergic neurons and long-descending projections to the raphe nucleus, as well as the periaqueductal gray matter of the mesencephalon, is critical for acupuncture analgesia [44–46]. The absence of midbrain/hypothalamus activation, by stimulation at the non-analgesic point, implies that the hypothalamus might characterize the central expression of acupuncture stimulation at the acupoint of prominent analgesic efficacy [47]. In addition, the hypothalamus may serve as one of the key neural substrates mediating the analgesic efficacy of acupuncture stimulation [48, 49].

Neuroanatomical study has shown that the cerebellum and hypothalamus are interconnected by direct hypothalamo-cerebellar and cerebello-hypothalamic projections, and by a multitude of indirect pathways [50]. The mutual connections between the cerebellum and hypothalamus may be part of the circuits through which the cerebellum modulates and coordinates a wide range of both somatic and non-somatic central nervous activities [51]. Although many nuclei have been found to be involved in electroacupuncture analgesia, it is not clear how they work together in electroacupuncture inhibition of persistent pain. The relevance of cerebellum activity to acupuncture stimulation mandates further exploration.

## Conclusions

In this study, we investigated the central representation of EA stimulation in nerve-injured rats. Enlarged and propagated activation patterns occurred after electrical stimulation of the hind paw, as it induced immediate reduction of neural activation after EA stimulation. These results suggest a correlation between the activation of S1 areas of brain cortices and neuropathic pain, providing novel indications that the analgesic effect of EA stimulation can be visualized in rat S1 cortex in a neuropathic pain animal model.

## Abbreviations

EA: Electroacupuncture
fMRI: Functional magnetic resonance imaging
PET: Positiron emission tomography
S1: Primary somatosensory cortex
SP9: Yinlingquan
ST36: Zusanli
VSD: Voltage-sensitive dye
